# ﻿Review of the spider genus *Amaurobius* (Araneae, Amaurobiidae) from China, with description of a new genus

**DOI:** 10.3897/zookeys.1245.152487

**Published:** 2025-07-15

**Authors:** Jin-Xia Kong, Yan-Nan Mu, Ben-Chao Zhu, Wei Lu, Zhi-Sheng Zhang, Lu-Yu Wang

**Affiliations:** 1 Key Laboratory of Eco-environments in Three Gorges Reservoir Region (Ministry of Education), School of Life Sciences, Southwest University, Chongqing 400715, China Southwest University Chongqing China; 2 Management Center of Daba Mountain National Nature Reserve, Chongqing 405909, China Management Center of Daba Mountain National Nature Reserve Chongqing China

**Keywords:** Amaurobiinae, description, mesh web spider, morphology, new combination, new species, taxonomy

## Abstract

This study reviews the Chinese representatives of *Amaurobius*, and a new genus, *Sinoamaurobius* Kong, Zhang & Wang, **gen. nov.**, is described to accommodate seven species, including three new species and four species transferred from *Amaurobius*: *Sinoamaurobiusguangwushanensis* (Wang, Irfan, Zhou & Zhang, 2023), **comb. nov.**, *S.songi* (Zhang, Wang & Zhang, 2018), **comb. nov.**, *S.spinatus* (Zhang, Wang & Zhang, 2018), **comb. nov.**, *S.wulongdongensis* (Wang, Irfan, Zhou & Zhang, 2023), **comb. nov.**, *S.baima* Kong, Zhang & Wang, **sp. nov.** (♀, Sichuan), *S.chengkou* Kong, Zhang & Wang, **sp. nov.** (♂♀, Chongqing) and *S.yintiaoling* Kong, Zhang & Wang, **sp. nov.** (♂♀, Chongqing). Additionally, we also describe three new *Amaurobius* species: *A.foping* Kong, Zhang & Wang, **sp. nov.** (♀, Shaanxi), *A.pingwu* Kong, Zhang & Wang, **sp. nov.** (♂♀, Sichuan) and *A.yushen* Kong, Zhang & Wang, **sp. nov.** (♂♀, Chongqing and Hubei). For all new species, morphological descriptions and photos of habitus and copulatory organs are provided.

## ﻿Introduction

At present, the family Amaurobiidae Thorell, 1869 comprises 202 species belonging to 26 genera distributed worldwide (WSC 2025). The family has never undergone a comprehensive, large-scale revision. Currently, 23 species from three genera are known in China, six of which belong to *Amaurobius*. This genus has been relatively well-studied in recent years, particularly due to key contributions such as Marusik et al. (2020), which resolved taxonomic confusion in *Amaurobius* species, especially those from the Caucasian and extra-Holarctic regions. Additionally, Bosmans (2021) addressed gaps in Mediterranean Amaurobiidae taxonomy, focusing on understudied areas (e.g., Algeria, Iberian Peninsula), and provided detailed morphological illustrations and distribution maps to aid future research. While studying this genus in China, we observed that these species cluster into two distinct size groups: smaller than 4.6 mm and larger than 8.6 mm. Further examination of their copulatory organs revealed clear morphological differences between these groups. Furthermore, species belonging to different size clusters have different habitat preferences.

Based on the above-mentioned details, we conclude that small-sized *Amaurobius* can be placed in a separate genus. The goal of this paper is a description of a new genus, and six new species belonging to the new genus and *Amaurobius*.

## ﻿Material and methods

All specimens were preserved in 75% ethanol and were examined, photographed and measured using a Leica M205A stereomicroscope equipped with a Leica DFC450 Camera and LAS software (ver. 4.6). Male palps were examined after they were dissected. Epigynes were cleared by immersing them in pancreatin for about 1 h (Álvarez-Padilla and Hormiga 2007). Eye sizes were measured as the maximum dorsal diameter. Leg measurements are shown as: total length (femur, patella and tibia, metatarsus, tarsus). All measurements are given in millimetres. Specimens examined here are deposited in the Collection of Spiders, School of Life Sciences, Southwest University, Chongqing, China (**SWUC**).

The following abbreviations are used in the text:

Somatic characters: **ALE**–anterior anterior lateral eye; **AME**–anterior median eye; **DTA**–dorsal tibial apophysis; **MOA**–median ocular area; **PLE**–posterior lateral eye; **PME**–posterior median eye; **RTA**–retrolateral tibial apophysis.

Male palp: **CF**–cymbial furrow; **Co**–conductor; **DTA**–dorsal tibial apophysis; **E**–embolus; **pb**–prolateral branch of dorsal tibial apophysis; **MA**–median apophysis; **RTA**–retrolateral tibial apophysis; **TA**–tegular apophysis.

Epigyne: **CD**–copulatory duct; **FD**–fertilization duct; **LT**–lateral tooth; **MP**–median plate; **S**–spermatheca.

## ﻿Taxonomy

### ﻿Family Amaurobiidae

Taxon classificationAnimaliaAraneaeAmaurobiidae

Thorell, 1869

9F8D49FC-7F8E-56D8-8708-8CFA910F1D5C

#### Remark.

The family Amaurobiidae comprises four subfamilies: Altellopsinae, Amaurobiinae, Arctobiinae and Ovtchinnikoviinae (Wheeler et al. 2017; Gorneau et al. 2023).

### ﻿Subfamily Amaurobiinae

Taxon classificationAnimaliaAraneaeAmaurobiidae

Thorell, 1869

2E66A0DB-A1C6-5161-9CBA-12F157E36D70

#### Remark.

The classification of Amaurobiinae remains unclear at present. However, it is certain that the genera *Amaurobius* C.L. Koch, 1837, *Callobius* Chamberlin, 1947, *Cybaeopsis* Strand, 1907, *Pimus* Chamberlin, 1947 and *Taira* Lehtinen, 1967 are assigned to the subfamily (Hu 2020).

### ﻿ Sinoamaurobius

Taxon classificationAnimaliaAraneaeAmaurobiidae

Kong, Zhang & Wang gen. nov.

A0BB9B1A-F6DD-53C7-9B56-828AC5B738DF

https://zoobank.org/231646C6-9114-4A17-85D6-BA51C099239B

#### Type species.

*Sinoamaurobiusyintiaoling* Kong, Zhang & Wang, sp. nov. from central China.

#### Etymology.

The generic name is a compound noun from the Latin *Sino* (meaning China) and *Amaurobius*. The gender is masculine.

#### Diagnosis.

*Sinoamaurobius* Kong, Zhang & Wang, gen. nov. resembles *Amaurobius* in having a similar short and curved embolus (Lu et al. 2024: 118, figs 1–6; Wang et al. 2023: 309, figs 1–4; Zhang et al. 2018: 365, figs 1–6), but can be distinguished by the small body size (3.2–4.76 mm) (vs 8.6–17.31 mm), the male palpal patella with a strong dorso-apical short spine (Figs 3C, E, 4C, E), except for *S.songi* lacking such spine (vs absent, Figs 6–8), large thumb-shaped or half-circular shape of the retrolateral tibial apophysis (RTA), originating near the base of the tibia (Figs 3D, 4D), except for *S.songi* originating near the middle part of the tibia (vs triangular or conical shape, originating near the middle part of the tibia, Figs 6D, 7D, 8D), median apophysis (MA) thin and long (Figs 3D, E, 4D, E) (vs thick and short, Figs 6–8).

**Figure 1.**
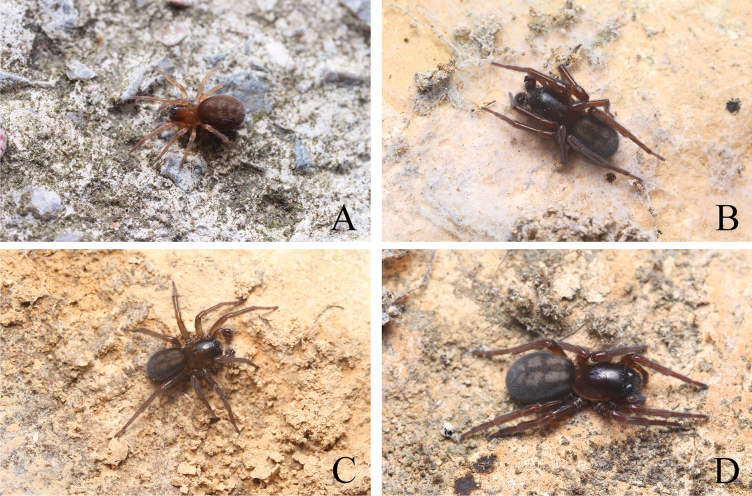
Photos of living spider. **A.***Sinoamaurobiusyintiaoling* Kong, Zhang & Wang, sp. nov., female; **B–D.***Amaurobiusyushen* Kong, Zhang & Wang, sp. nov. from Yintiaoling: **B, C.** Male; **D.** Female. Photographed by Qian-Le Lu.

#### Description.

**Figure 2.**
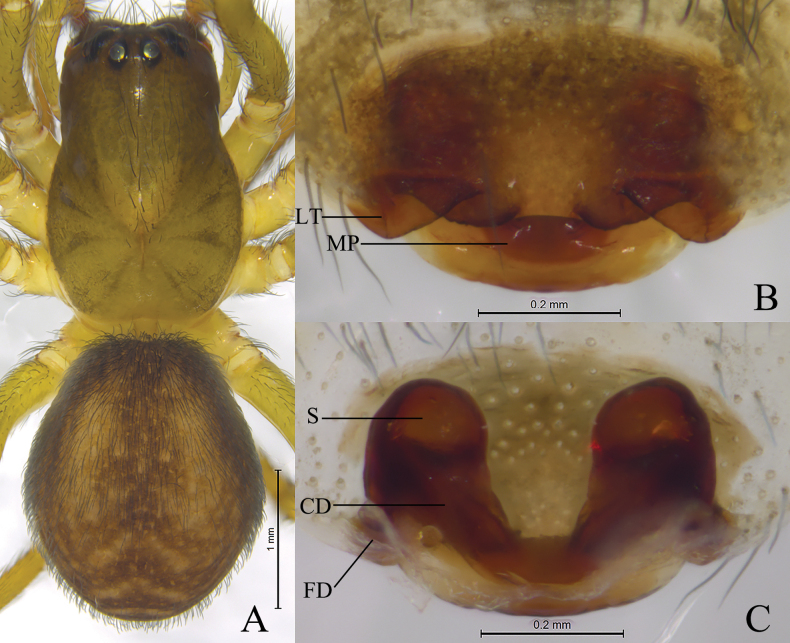
*Sinoamaurobiusbaima* Kong, Zhang & Wang, sp. nov., holotype female. **A.** Female habitus, dorsal view; **B.** Epigyne, ventral view; **C.** Vulva, dorsal view.

**Figure 3.**
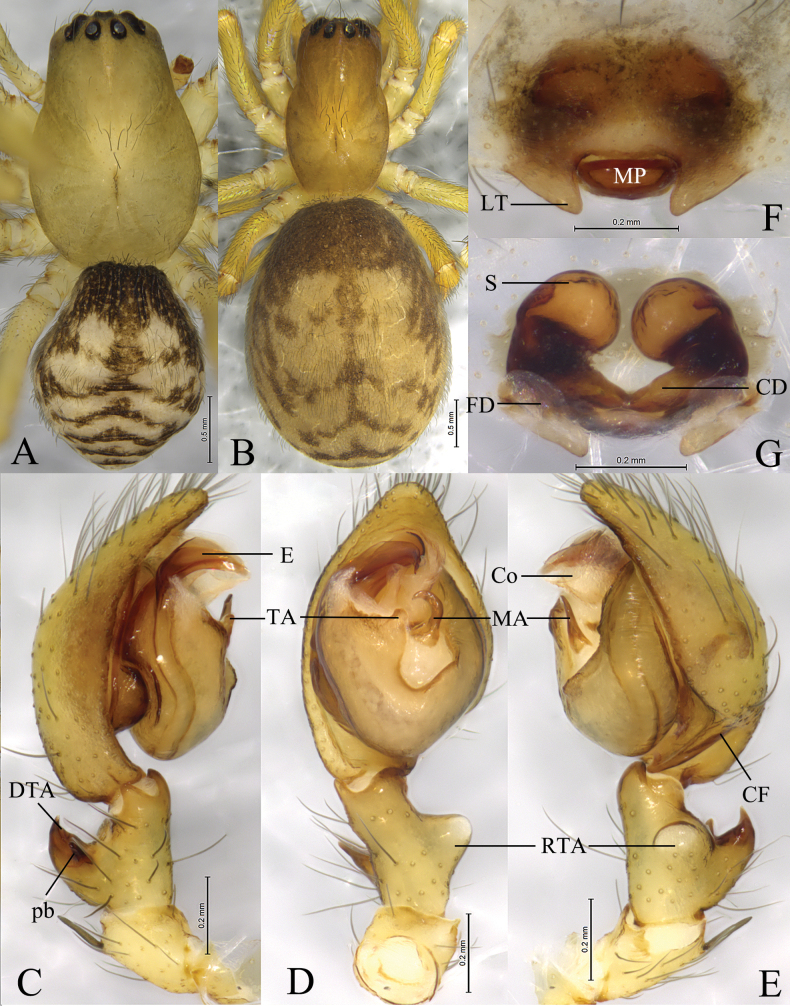
*Sinoamaurobiuschengkou* Kong, Zhang & Wang, sp. nov. **A, C–E.** Holotype male; **B, F, G.** Paratype female. **A.** Male habitus, dorsal view; **B.** Female habitus, dorsal view; **C.** Left male palp, prolateral view; **D.** Same, ventral view; **E.** Same, retrolateral view; **F.** Epigyne, ventral view; **G.** Vulva, dorsal view.

**Figure 4.**
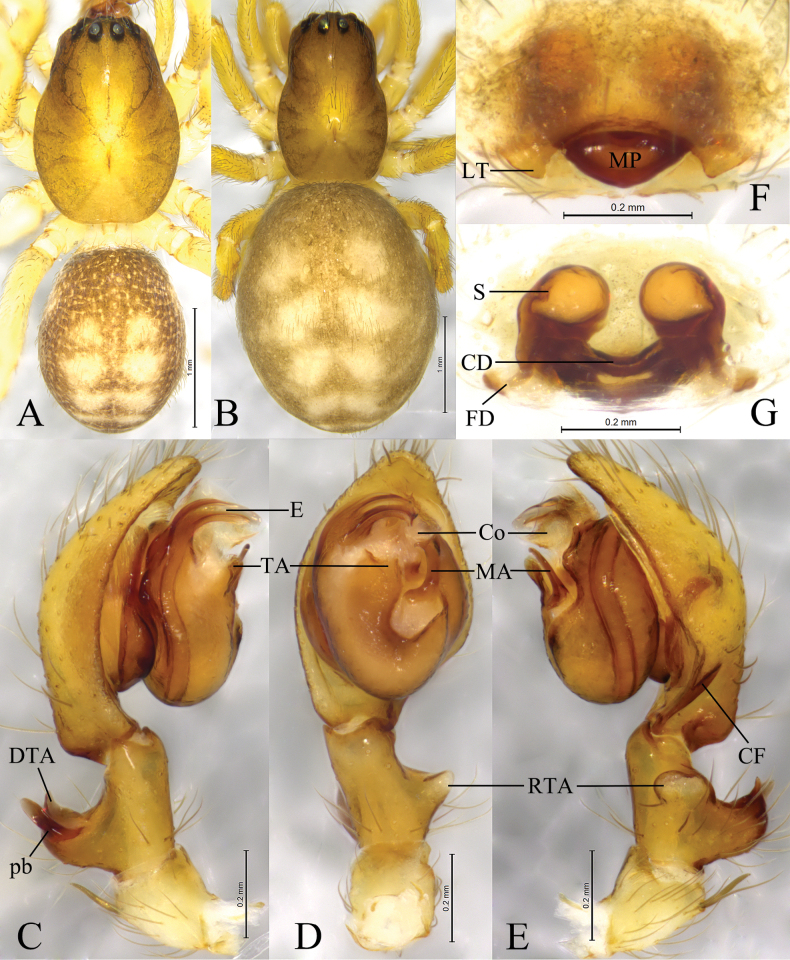
*Sinoamaurobiusyintiaoling* Kong, Zhang & Wang, sp. nov. **A, C–E.** Holotype male; **B, F, G.** Paratype female. **A.** Male habitus, dorsal view; **B.** Female habitus, dorsal view; **C.** Left male palp, prolateral view; **D.** Same, ventral view; **E.** Same, retrolateral view; **F.** Epigyne, ventral view; **G.** Vulva, dorsal view.

Total length 3.20–4.76. Carapace yellowish. Eyes region black (Figs 2A, 3A, B, 4A, B). Chelicerae with 3 or 5 teeth on both margins. Legs yellowish. Leg formula in both sexes: 1423. Opisthosoma oval, dorsum yellowish, with six brown chevrons extending posteriorly, venter yellowish brown.

***Male palp*** (Figs 3C–E, 4C–E): Femur almost as long as cymbium; patella with strong dorso-apical short spine; tibia with large thumb-shaped retrolateral apophysis (RTA), dorsal tibial apophysis (DTA) subequal to length of the tibia, hook-like, slightly curved dorsally, with prolateral branch (pb) triangular. Cymbium with distinct fold (CF) located retro-proximally. Tegulum widest in middle part. Sperm duct visible in prolateral and retrolateral view. Median apophysis (MA) sclerotized, hook-like, located near center of tegulum. Conductor membranous and sheet-like. Tegular apophysis (TA) present. Embolus originating prolaterally, short, flat and sharply pointed, with thin apex.

***Epigyne*** (Figs 2B, C, 3B–D, 4F, G): Epigynal plate oval, about 2 times wider than long; with distinct median plate (MP) of variable shape and lateral teeth (LT); copulatory ducts (CD) U or V-shaped, located between spermathecae and connected to each other; spermathecae (S) globular, separated by diameter; fertilization ducts (FD) originating posteriorly, slightly curved, directed laterally.

#### Biology.

Inhabit leaf litter on evergreen forest floors.

#### Composition.

*Sinoamaurobiusbaima* Kong, Zhang & Wang, sp. nov., *S.chengkou* Kong, Zhang & Wang, sp. nov., *S.guangwushanensis* (Wang, Irfan, Zhou & Zhang, 2023), comb. nov., *S.songi* (Zhang, Wang & Zhang, 2018), comb. nov., *S.spinatus* (Zhang, Wang & Zhang, 2018), comb. nov., *S.wulongdongensis* comb. nov. (Wang, Irfan, Zhou & Zhang, 2023) and *S.yintiaoling* Kong, Zhang & Wang, sp. nov.

#### Distribution.

China (Chongqing, Sichuan, Shaanxi) from the south slope of the Qinling Mountains to the eastern extension of the Hengduan Mountains (Fig. 9).

### ﻿ Sinoamaurobius baima

Taxon classificationAnimaliaAraneaeAmaurobiidae

Kong, Zhang & Wang sp. nov.

96EAA8EC-02D2-52D4-91AA-0075F2E272B4

https://zoobank.org/51947EFD-735B-4249-B07A-6FDC6C330D15

Figs 2 , 9 白马华暗蛛

#### Type material.

***Holotype*** • ♀ (SWUC-T-AM-21-01): **China**, Sichuan Prov., Pingwu Co., Baima Tibetan Vill.; 32°42'46″N, 104°22′37″E, elev. 1800 m; 24–25.9.2019; L.Y. Wang et al. leg.; ***Paratype*** • 1♀ (SWUC-T-AM-21-02): same locality as holotype; 16.9.2021; L.Y. Wang et al. leg.

#### Etymology.

The specific name is derived from the type locality; noun in apposition.

#### Diagnosis.

The new species resembles *S.guangwushanensis* (Wang et al. 2023: 308, figs 1D, E, 2B, F, G) in having similar lateral teeth (LT) and spermathecae (S), but can be distinguished by the U-shaped copulatory ducts (Fig. 2C) (vs transverse).

#### Description.

**Female** holotype (Fig. 2A) total length 4.45. Carapace 2.16 long, 1.44 wide; opisthosoma 2.32 long, 1.64 wide. Carapace yellowish green. Eye sizes and interdistances: AME 0.07, ALE 0.13, PME 0.13, PLE 0.12; AME–AME 0.07, AME–ALE 0.08, PME–PME 0.12, PME–PLE 0.15, ALE–PLE 0.07. MOA 0.33 long, front width 0.20, back width 0.37. Clypeus height 0.17. Chelicerae with 3 promarginal and 3 retromarginal teeth. Leg measurements: I 5.63 (1.57, 2.01, 1.23, 0.82); II 4.34 (1.22, 1.50, 0.98, 0.64); III 3.59 (1.12, 1.08, 0.89, 0.50); IV 4.90 (1.39, 1.72, 1.21, 0.58).

***Epigyne*** (Fig. 2B, C). Epigynal plate 1.6 times wider than long. Median plate (MP) about 2 times wider than long; lateral teeth (LT) sheet-shaped; copulatory ducts (CD) U-shaped, located between spermathecae and connected to each other. Spermathecae (S) globular, separated by diameter. Fertilization ducts (FD) originating posteriorly, slightly curved, directed laterally.

**Male** unknown.

#### Variation.

Females (*N* = 2) total length 4.45–4.50.

#### Distribution.

Known only from the type locality, Sichuan, China (Fig. 9).

### ﻿ Sinoamaurobius chengkou

Taxon classificationAnimaliaAraneaeAmaurobiidae

Kong, Zhang & Wang sp. nov.

4DC6FE08-F114-58D3-B108-5EE8639E2F0C

https://zoobank.org/7CFFCF90-C73D-414C-B551-B75C2C7B2BE9

Figs 3 , 9 城口华暗蛛

#### Type material.

***Holotype*** • ♂ (SWUC-T-AM-22-01): **China**, Chongqing Munic., Chengkou Co., Longtian Township, Fenglian Vill. Nacaigou; 32°3′3″N, 108°42′45″E, elev. 1260 m; 2.11.2022; Z.S. Zhang et al. leg. ***Paratypes*** (SWUC-T-AM-22-02 to 09) (8♀): • 3♀ (SWUC-AM-22-02 to 04), same locality as holotype; 17.05.2025; L.Y. Wang leg.; • 5♀ (SWUC-AM-22-05 to 09), Fenglian Vill., Tangjiayuanzi; 32°2′32″N, 108°44′52″E, elev. 1315 m; 17.05.2025; L.Y. Wang leg.

#### Etymology.

The specific name is derived from the type locality; noun in apposition.

#### Diagnosis.

Male of the new species resembles those of *S.wulongdongensis* (Wang et al. 2023: 308, figs 3A–C, 4A, C–E) in having a similar embolus and retrolateral tibial apophysis, but can be distinguished by the dorsal tibial apophysis (DTA) hook-like (Fig. 3B, D) (vs tip wavy); apex of the tegular apophysis (TA) bifurcated in ventral view (Fig. 3C) (vs not bifurcated). The female of the new species resembles those of *S.yintiaoling* Kong, Zhang & Wang, sp. nov. (Fig. 4) in having similar globular spermathecae, but can be distinguished by the trapezoid median plate (ML) (Fig. 3F) (vs triangular); copulatory ducts (CD) V-shaped (Fig. 3G) (vs U-shaped); fertilization ducts (FD) originating posteriorly, slightly curved, no obvious outward extension (vs with distinct extension outward before bending).

#### Description.

**Male** holotype (Fig. 3A) total length 3.46. Carapace 1.93 long, 1.31 wide; opisthosoma 1.57 long, 1.34 wide. Carapace yellowish. Eye sizes and interdistances: AME 0.05, ALE 0.13, PME 0.10, PLE 0.10; AME–AME 0.05, AME–ALE 0.07, PME–PME 0.11, PME–PLE 0.12, ALE–PLE 0.05. MOA 0.23 long, front width 0.14, back width 0.29. Clypeus height 0.12. Chelicerae with 3 promarginal and 5 retromarginal teeth. Leg measurements: I 7.69 (2.09, 2.75, 1.92, 0.93); II 5.19 (1.41, 1.73, 1.29, 0.76); III 4.38 (1.27, 1.43, 1.05, 0.63); IV 5.47 (1.56, 1.93, 1.42, 0.56). Opisthosoma dorsum brownish, with 2 pairs of yellow spots in anterior part, 3 pairs of chevrons in posterior part, and venter brownish.

***Palp*** (Fig. 3B–D). Tibia about 2 times longer than wide, with dorsal apophysis (DTA) equivalent to 2/3 length of tibia, hook-like, slightly curved dorsally, prolateral branch (pb) triangular. Cymbium 1.5 times longer than tibia, with retrolateral angular projection. Tegulum widest in middle part. Tegular apophysis (TA) thumb-shaped. Sperm duct visible in prolateral and retrolateral view. Median apophysis (MA), hook-like, located near center of tegulum. Embolus originating prolaterally, flat and sharply pointed, with thin apex.

**Female** paratype (SWUC-T-AM-22-02, Fig. 3B) total length 4.76. Carapace 1.87 long, 1.12 wide; opisthosoma 2.87 long, 2.18 wide. Eye sizes and interdistances: AME 0.04, ALE 0.12, PME 0.10, PLE 0.12; AME–AME 0.05, AME–ALE 0.07, PME–PME 0.12, PME–PLE 0.14, ALE–PLE 0.05. MOA 0.17 long, front width 0.33, back with 0.30. Clypeus height 0.11. Chelicerae as in male. Leg measurements: I 4.99 (1.42, 1.74, 1.14, 0.69); II 3.38 (1.01, 1.15, 0.74, 0.48); III 3.39 (1.02, 1.15, 0.74, 0.48); IV 4.57 (1.37, 1.57, 1.05, 0.58). Opisthosomal pattern as in male.

***Epigyne*** (Fig. 3F, G). Epigynal plate 1.6 times wider than long. Median plate (MP) trapezoidal, 2.25 times wider than long. Lateral teeth (LT) triangular, blunt. Copulatory ducts (CD) V-shaped. Spermathecae (S) globular, closely spaced. Fertilization ducts (FD) originating posteriorly, slightly curved, no obvious outward extension.

#### Variation.

Females (*N* = 8) total length 3.52–4.76.

#### Distribution.

Known only from the type locality, Chongqing, China (Fig. 9).

### ﻿ Sinoamaurobius guangwushanensis

Taxon classificationAnimaliaAraneaeAmaurobiidae

(Wang, Irfan, Zhou & Zhang, 2023) comb. nov.

117013B4-E728-5649-9D18-6221C8E39C87

Amaurobius guangwushanensis Wang et al., 2023: 308, figs 1A–E, 2A–E (♂♀ from Nanjiang Co. of Sichuan, deposited in SWUC, examined).

#### Diagnosis and description.

See Wang et al. (2023).

#### Distribution.

Known only from the type locality, Sichuan of China (Fig. 9).

#### Remark.

This species is transferred to *Sinoamaurobius* Kong, Zhang & Wang, gen. nov. due to the similarity in its copulatory organs to those of the generotype.

### ﻿ Sinoamaurobius songi

Taxon classificationAnimaliaAraneaeAmaurobiidae

(Zhang, Wang & Zhang, 2018) comb. nov.

9A54AC16-AB3A-5D61-BFA0-29B727694E78

Amaurobius songi Zhang, Wang & Zhang, 2018: 367, figs 1A–E, 2A–G, 3A–D (♂♀ from Chongzhou City of Sichuan, deposited in SWUC, examined).

#### Description and diagnosis.

See Zhang et al. (2018).

#### Distribution.

Known only from the type locality, Sichuan, China (Fig. 9).

#### Remark.

This species is transferred to *Sinoamaurobius* Kong, Zhang & Wang, gen. nov. due to the similarity in its copulatory organs to those of the generotype.

### ﻿ Sinoamaurobius spinatus

Taxon classificationAnimaliaAraneaeAmaurobiidae

(Zhang, Wang & Zhang, 2018) comb. nov.

F7882648-16A9-5A92-91B7-C705702CB85D

Amaurobius spinatus Zhang, Wang & Zhang, 2018: 367, figs 4A–E, 5A–G, 6A–D (♂♀ from Chengkou Co. of Chongqing, deposited in SWUC, examined).

#### Description and diagnosis.

See Zhang et al. (2018).

#### Distribution.

Known only from the type locality, Chongqing of China (Fig. 9).

#### Remark.

This species is transferred to *Sinoamaurobius* Kong, Zhang & Wang, gen. nov. based on the similarity of its copulatory organs to those of the generotype.

### ﻿ Sinoamaurobius wulongdongensis

Taxon classificationAnimaliaAraneaeAmaurobiidae

(Wang, Irfan, Zhou & Zhang, 2023) comb. nov.

E0260D4E-1A92-52AD-A9ED-5BCDB9F1C740

Amaurobius wulongdongensis Wang et al. 2023: 308, figs 3A–E, 4A–E (♂♀ from Lueyang Co. of Shaanxi deposited in SWUC, examined).

#### Description and diagnosis.

See Wang et al. (2023).

#### Distribution.

Known only from the type locality, Shaanxi, China (Fig. 9).

#### Remark.

This species is transferred to the new genus *Sinoamaurobius* based on the similarity of its copulatory organs to those of the type species of the genus.

### ﻿ Sinoamaurobius yintiaoling

Taxon classificationAnimaliaAraneaeAmaurobiidae

Kong, Zhang & Wang sp. nov.

D01DB300-4258-5020-A691-A98624159101

https://zoobank.org/F71EA162-B209-4AE3-8945-FA16FA6AEDAC

Figs 1A , 4 , 9 阴条岭华暗蛛

#### Type material.

***Holotype*** • ♂ (SWUC-T-AM-23-01): **China**, Chongqing Munic., Wuxi Co., Yintiaoling Nature Reserve, Hongqi Station, nr Shuangtong Reservoir; 31°31′22″N, 109°49′33″E, elev. 1209 m; 30.11.2021; Z.S. Zhang et al. leg.; ***Paratypes*** (1♂16♀): • 1♂ (SWUC-T-AM-23-02), same data as holotype; • 11♀ (SWUC-T-AM-23-03 to 13), nr Shuangtong Reservoir; 31°31′29″N, 109°49′30″E, elev. 1261 m; 10.4.2022; Z.S. Zhang et al. leg.; • 4♀ (SWUC-T-AM-23-14 to 17), Shuangtong Reservoir; 31°31′22″N, 109°49′36″E, elev. 1194 m; 10.4.2022; Z.S. Zhang et al. leg.; • 1♀ (SWUC-T-AM-23-18), nr Shuangtong Reservoir; 31°31′25″N, 109°49′32″E, elev. 1258 m; 23.9.2022; L.Y. Wang et al. leg.

#### Etymology.

The specific name is derived from the type locality; noun in apposition.

#### Diagnosis.

The new species resembles *S.spinatus* (Zhang et al. 2018: 367, figs 4A–E, 5A–G, 6A–D) in having a similar conductor, embolus, and spermathecae. Males of the new species can be distinguished by the triangular retrolateral tibial apophysis (RTA) in ventral view (Fig. 4D) (vs somewhat rectangular); prolateral branch of the dorsal tibial apophysis (pb) hook-shaped in prolateral view (Fig. 4C) (vs thumb-shaped); apex of the tegular apophysis (TA) bifurcated in ventral view (Fig. 4D) (vs not bifurcated); median apophysis (MA) doorknob-shaped (Fig. 4D) (vs hook-shaped). The females can be separated by the median plate (MP) somewhat triangular (Fig. 4F) (vs oval); lateral teeth (LT) 1/3 length of median plate with blunt tip (Fig. 4F) (vs about half the length of median plate, with tapering tip); spermathecae spaced by about half of the radius (Fig. 4G) (vs less than half radius of the spermathecae).

#### Description.

**Male** holotype (Fig. 4A) total length 3.52. Carapace 1.74 long, 1.23 wide; opisthosoma 1.63 long, 1.27 wide. Carapace yellowish. Eye sizes and interdistances: AME 0.05, ALE 0.10, PME 0.10, PLE 0.10; AME–AME 0.04, AME–ALE 0.06, PME–PME 0.10, PME–PLE 0.11, ALE–PLE 0.04. MOA 0.26 long, front width 0.15, back width 0.29. Clypeus height 0.11. Chelicerae with 4 promarginal and 4 retromarginal teeth. Leg measurements: I 5.79 (1.48, 2.06, 1.42, 0.83); II 4.23 (1.20, 1.40, 1.03, 0.60); III 3.66 (1.11, 1.06, 0.95, 0.54); IV 4.69 (1.42, 1.54, 1.05, 0.68). Opisthosoma dorsum yellowish, with six white chevrons extending posteriorly, and venter yellowish.

***Palp*** (Fig. 4C–E). Femur almost as long as cymbium. Tibia with large thumb-shaped RTA, 2/3 length of tibia, originating near base of tibia. Dorsal apophysis (DTA) large about half of tibia length, slightly curved dorsally, and prolateral branch of dorsal apophysis (pb) hook-like. Cymbium 1.5 times longer than tibia, with retrolateral angular projection. Tegular apophysis (TA) triangular in ventral view. Median apophysis (MA) hook-like, located near center of tegulum. Embolus originating prolaterally, short, flat and sharply pointed, with thin tip.

**Female** paratype (SWUC-T-AM-23-03, Fig. 4B) total length 4.42. Carapace 1.81 long, 1.14 wide; opisthosoma 2.65 long, 2.11 wide. Eye sizes and interdistances: AME 0.06, ALE 0.11, PME 0.11, PLE 0.11; AME–AME 0.04, AME–ALE 0.07, PME–PME 0.11, PME–PLE 0.15, ALE–PLE 0.06. MOA 0.33 long, front width 0.16, back with 0.31. Clypeus height 0.11. Chelicerae as in male. Leg measurements: I 4.4 (1.31, 1.51, 0.95, 0.63); II 3.54 (1.07, 1.20, 0.75, 0.52); III 2.95 (0.94, 0.96, 0.61, 0.44); IV 4.03 (1.22, 1.32, 1.01, 0.48). Opisthosoma as in male.

***Epigyne*** (Fig. 4F, G). Epigynal plate 1.6 times wider than long. Median plate (MP) triangular, almost 2 times wider than long; lateral teeth (LT) triangular, with blunt tips. Copulatory ducts (CD) U-shaped. Spermathecae (S) globular, spaced by about half of radius. Fertilization ducts (FD) originating posteriorly.

#### Variation.

Male (*N* = 2) total length 3.53–3.63, females (*N* = 16) total length 3.51–4.48.

#### Distribution.

Known only form the type locality, Yintiaoling Natural Reserve, Chongqing, China (Fig. 9).

### ﻿Genus Amaurobius

Taxon classificationAnimaliaAraneaeAmaurobiidae

C.L. Koch, 1837

9FA5B5BC-E254-5C9F-85AA-83931CC94953

#### Type species.

*Araneaatrox* De Geer, 1778 (considered a junior synonym of *A.fenestralis* Ström, 1768) from Sweden.

#### Distribution.

It has disjunct distribution and is known in the West Palearctic east to Iran, in Eastern China (Chongqing, Hubei, Sichuan, Shaanxi) and in the Nearctic (from Canada to northern Mexico, all species, except one, are restricted to the western part of the Nearctic).

### ﻿ Amaurobius foping

Taxon classificationAnimaliaAraneaeAmaurobiidae

Kong, Zhang & Wang sp. nov.

3C094E10-BA0F-599E-B253-3765E875A573

https://zoobank.org/1DA58E23-B0B4-4295-9AE4-421D8171DFF7

Figs 5 , 9 佛坪暗蛛

#### Type material.

***Holotype*** • ♀ (SWUC-T-AM-24-01): **China**, Shaanxi Prov., Foping Co., Xiongmao Valley; 33°40′21″N, 107°57′37″E, elev. 1332 m; 17.5.2018; Z.S. Zhang et al. leg.

#### Etymology.

The specific name is derived from the type locality; noun in apposition.

#### Diagnosis.

**Figure 5.**
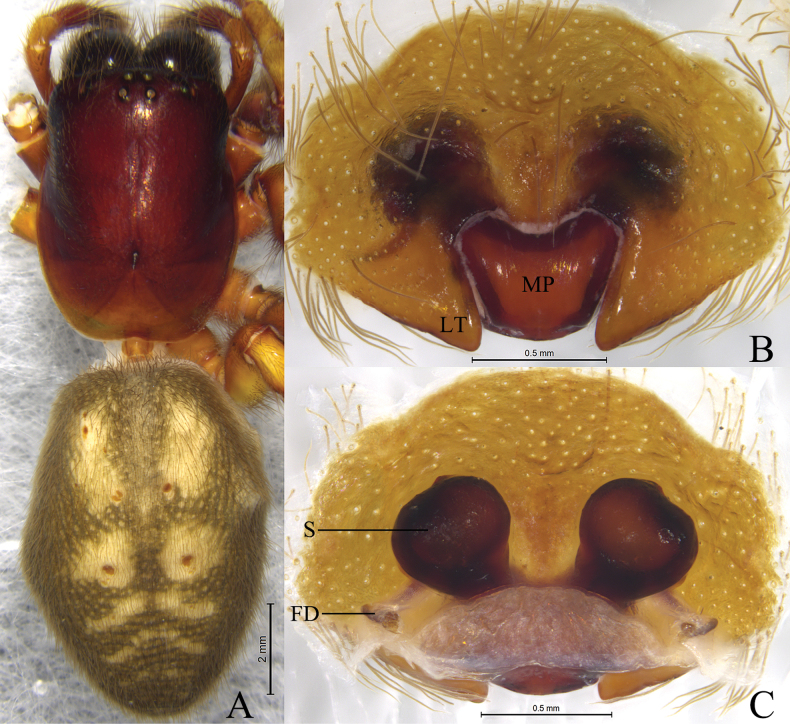
*Amaurobiusfoping* Kong, Zhang & Wang, sp. nov., holotype female. **A.** Female habitus, dorsal view; **B.** Epigyne, ventral view; **C.** Vulva, dorsal view.

The new species resembles *A.yaan* Lin & Li, 2024 (Lu et al. 2024: 121, figs 4A, B, 6A, B, 8G, H), in having similarly triangular lateral teeth (LT) of the epigyne, but can be distinguished by the median plate (MP) longer than wide (Fig. 5B) (vs as wide as long) and the copulatory ducts (CD) being inconspicuous (Fig. 5C) (vs conspicuous).

#### Description.

**Female** holotype (Fig. 5A) total length 14.22. Carapace 6.0 long, 4.43 wide, opisthosoma 7.89 long, 5.37 wide. Carapace reddish. Eye sizes and interdistances: AME 0.25, ALE 0.27, PME 0.18, PLE 0.24; AME–AME 0.17, AME–ALE 0.45, PME–PME 0.38, PME–PLE 0.74, ALE–PLE 0.22. MOA 0.73 long, front width 0.67, back width 0.82. Clypeus height 0.36. Chelicerae with 4 promarginal and 4 retromarginal teeth. Legs brownish. Leg measurements: I 9.18 (3.72, 3.15, 1.47, 0.84); II 11.56 (3.48, 3.95 2.63, 1.50); III 10.15 (3.26, 3.17, 2.37, 1.35); IV 14.07 (3.79, 5.34, 3.16, 1.78). Opisthosoma oval, dorsum brownish, with 2 pairs of yellow spots in anterior part, 3 pairs of chevrons in posterior part, venter brownish.

***Epigyne*** (Fig. 5B, C). Epigynal plate 1.4 times wider than long, median plate (MP) trapezoidal, apparently anterior margin excavated medially, wider than long; lateral teeth (LT) triangular, blunt. Spermathecae (S) globular, spaced by about half of radius, copulatory ducts (CD) inconspicuous. Fertilization ducts (FD) originating posteriorly.

**Male** unknown.

#### Distribution.

Known only from the type locality, Shaanxi, China (Fig. 9).

### ﻿ Amaurobius pingwu

Taxon classificationAnimaliaAraneaeAmaurobiidae

Kong, Zhang & Wang sp. nov.

BBE880E1-8B4A-599E-A0A7-60FE2B9713DA

https://zoobank.org/D0469CB7-29D9-4A3D-9D4E-500B826F7672

Figs 6 , 9 平武暗蛛

#### Type material.

***Holotype*** • ♂ (SWUC-T-AM-25-01): **China**, Sichuan Prov., Pingwu Co., Baima Tibetan Vill.; 32°42′46″N, 104°22′37″E, elev. 1801 m; 24–25.9.2019; L.Y. Wang et al. leg.; ***Paratypes***: • 6♂3♀ (SWUC-T-AM-25-02 to 10), same data as holotype.

#### Etymology.

The specific name is derived from the type locality; noun in apposition.

#### Diagnosis.

**Figure 6.**
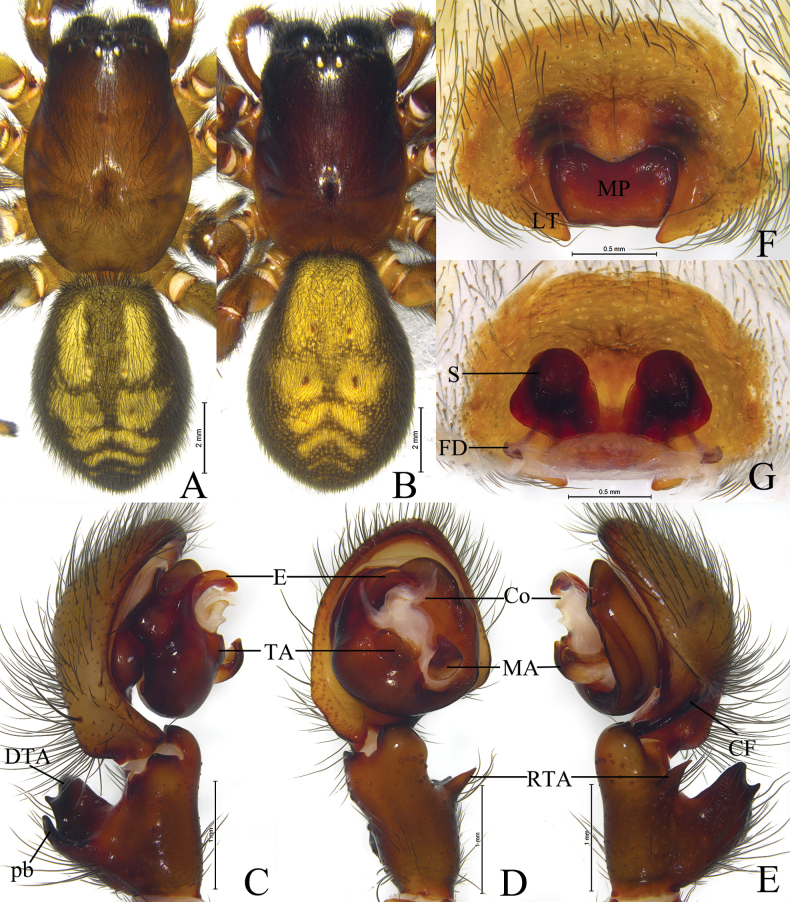
*Amaurobiuspingwu* Kong, Zhang & Wang, sp. nov. **A, C–E.** Holotype male; **B, F, G.** Paratype female. **A.** Male habitus, dorsal view; **B.** Female habitus, dorsal view; **C.** Left male palp, prolateral view; **D.** Same, ventral view; **E.** Same, retrolateral view; **F.** Epigyne, ventral view; **G.** Vulva, dorsal view.

The new species resembles *A.danba* Lin & Li, 2024 (Lu et al. 2024: 121, figs 1A, B, 2A, B, 3A, B, 8A–D) in having similar male palpal tibia and embolus, and females have similar lateral teeth (LT). Males of the new species can be recognized by having conical retrolateral tibial apophysis (Fig. 6D) (vs triangular); females can be separated by the anterior margin of median plate (MP) with distinct excision (Fig. 6F) (vs both anterior and posterior margins with excision).

#### Description.

**Male** holotype (Fig. 6A), total length 13.22. Carapace 7.04 long, 4.53 wide; opisthosoma 6.14 long, 4.31 wide. Carapace reddish brown. Eye sizes and interdistances: AME 0.21, ALE 0.29, PME 0.18, PLE 0.22; AME–AME 0.19, AME–ALE 0.42, PME–PME 0.36, PME–PLE 0.68, ALE–PLE 0.14. MOA 0.69 long, front width 0.57, back width 0.75. Clypeus height 0.31. Chelicerae reddish brown, with 5 promarginal and 4 or 5 retromarginal teeth. Legs yellowish brown. Leg measurements: I 17.21 (4.80, 5.97, 4.02, 2.42); II 15.66 (4.55, 5.49, 3.63, 1.99); III 14.13 (4.31, 4.87, 3.86, 1.59); IV 17.22 (4.94, 6.45, 3.94, 1.89). Opisthosoma oval, dorsum grass greenish, with 2 pairs of yellow spots in anterior part, 3 pairs of chevrons in posterior part, and venter greenish.

***Pale*** (Fig. 6C–E). Tibia with conical RTA, originating near middle part of tibia. DTA large, with 3 branches: prolateral one (pb) triangle-shaped, longer than wide, retrolateral branch larger than other, and intermediate branch spine-like. Cymbium as long as tibia, with retrolateral angular projection. Tegulum widest in middle part. Tegular apophysis (TA) slightly raised. Sperm duct visible in retrolateral view. Median apophysis (MA) hook-like, located near center of tegulum. Conductor membranous and sheet-like. Embolus originating prolaterally, short, flat and sharply pointed.

**Female** paratype (SWUC-T-AM-25-02, Fig. 6B) total length 13.95. Carapace 6.75 long, 4.73 wide; opisthosoma 7.50 long, 5.05 wide. Eye sizes and interdistances: AME 0.19, ALE 0.27, PME 0.19, PLE 0.24; AME–AME 0.19, AME–ALE 0.52, PME–PME 0.46, PME–PLE 0.78, ALE–PLE 0.20. MOA 0.74 long, front width 0.64, back with 0.83. Clypeus height 0.33. Legs reddish brown. Leg measurements: I 14.98 (4.27, 5.45, 3.16, 2.10); II 13.36 (4.03, 4.81, 2.72, 1.80); III 11.67 (3.33, 4.40, 2.57, 1.37); IV 14.61 (4.37, 5.30, 3.36, 1.58). Chelicerae with 5 promarginal and 4 or 5 retromarginal teeth. Opisthosoma same as in male.

***Epigyne*** (Fig. 6F, G). Epigynal plate 1.4 times wider than long. Median plate (MP) roughly trapezoidal, with excision on anterior margin; lateral teeth (LT) triangular, pointed. Copulatory ducts (CD) transverse, located between spermathecae and connected to each other. Spermathecae (S) globular, spaced by about half of radius. Fertilization ducts (FD) originating posteriorly.

#### Variation.

Male (*N* = 7) total length 12.53–13.22, females (*N* = 3) total length 13.95–17.31.

#### Distribution.

Known only from the type locality, Sichuan, China (Fig. 9).

### ﻿ Amaurobius yushen

Taxon classificationAnimaliaAraneaeAmaurobiidae

Kong, Zhang & Wang sp. nov.

488618F9-3FC5-5B23-B2D2-C97696364156

https://zoobank.org/9582B37A-4B53-4750-BEBD-6E75BD4E1DA9

**Figure 7.**
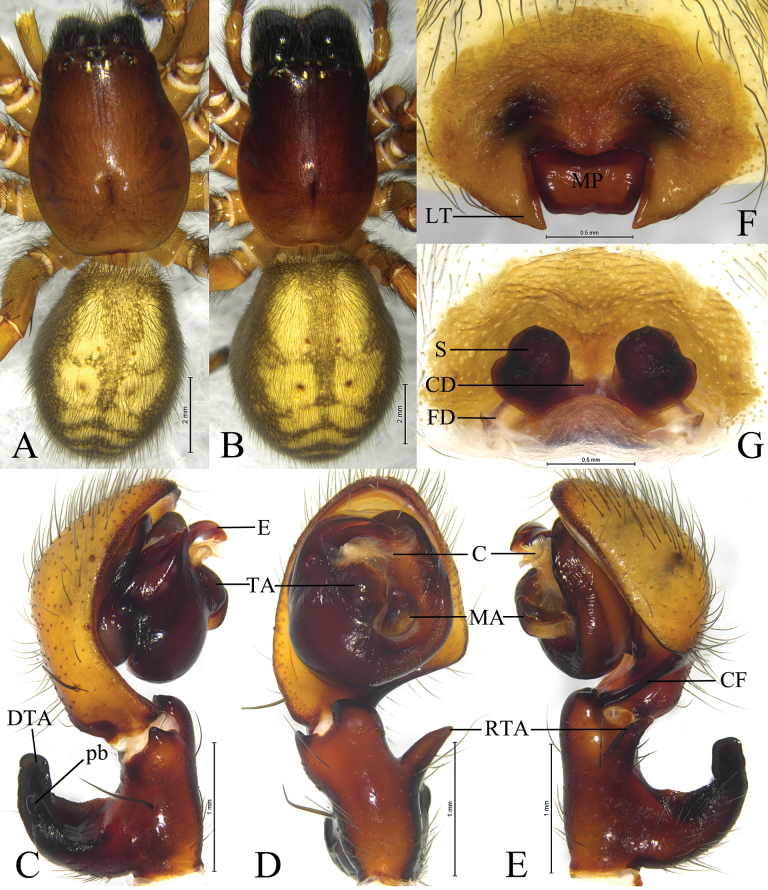
*Amaurobiusyushen* Kong, Zhang & Wang, sp. nov. from Yintiaoling. **A, C–E.** Holotype male; **B, F, G.** Paratype female. **A.** Male habitus, dorsal view; **B.** Female habitus, dorsal view; **C.** Left male palp, prolateral view; **D.** Same, ventral view; **E.** Same, retrolateral view; **F.** Epigyne, ventral view; **G.** Vulva, dorsal view.

**Figure 8.**
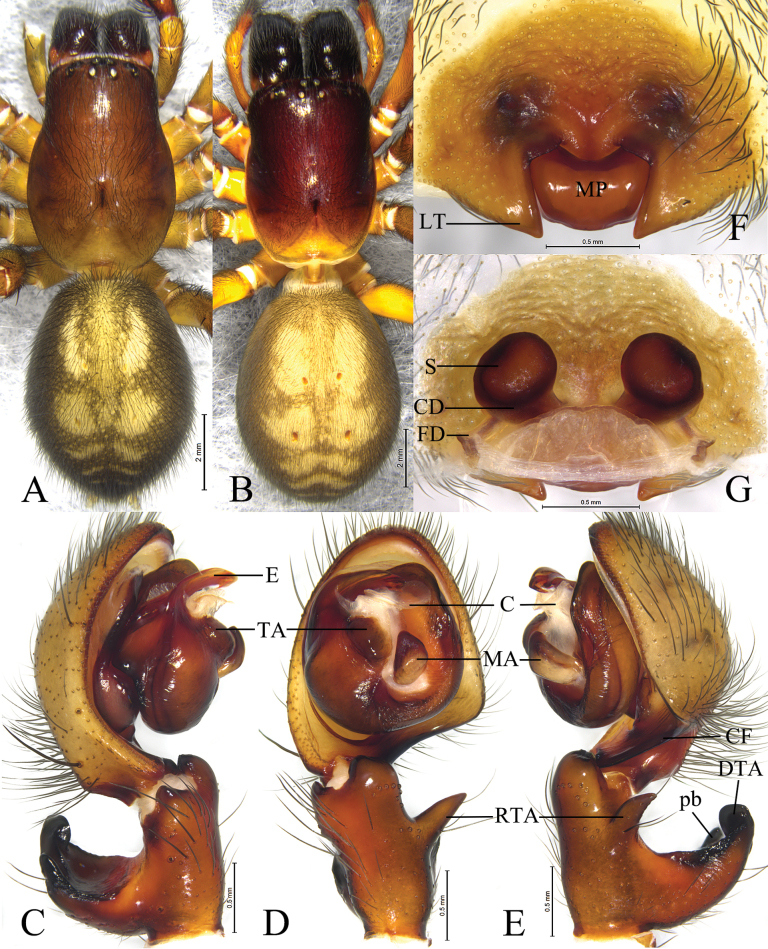
*Amaurobiusyushen* Kong, Zhang & Wang, sp. nov. from Chengkou. **A, C–E.** Paratype male; **B, F, G.** Paratype female. **A.** Male habitus, dorsal view; **B.** Female habitus, dorsal view; **C.** Left male palp, prolateral view; **D.** Same, ventral view; **E.** Same, retrolateral view; **F.** Epigyne, ventral view; **G.** Vulva, dorsal view.

**Figure 9.**
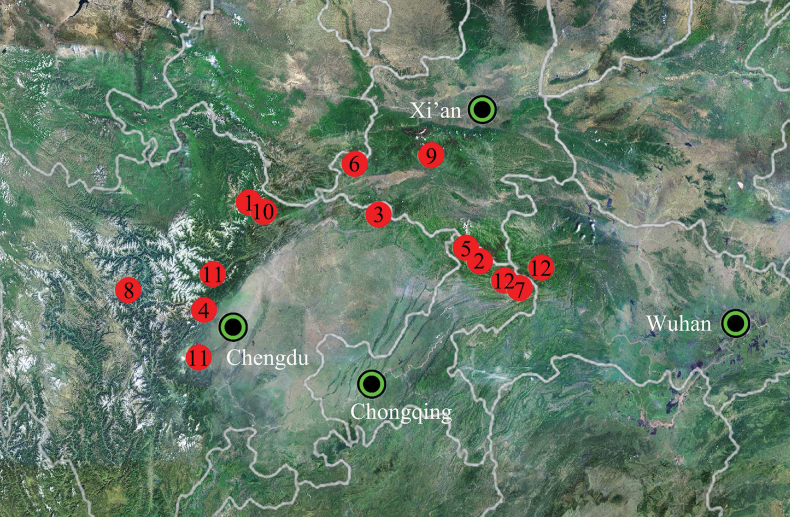
Distribution of five *Amaurobius* and seven *Sinoamaurobius* Kong, Zhang & Wang, gen. nov. species in China. **1.***Sinoamaurobiusbaima* Kong, Zhang & Wang, sp. nov.; **2.***S.chengkou* Kong, Zhang & Wang, sp. nov.; **3.***S.guangwushanensis* (Wang, Irfan, Zhou & Zhang, 2023); **4.***S.songi* (Zhang, Wang & Zhang, 2018); **5.***S.spinatus* (Zhang, Wang & Zhang, 2018); **6.***S.wulongdongensis* (Wang, Irfan, Zhou & Zhang, 2023); **7.***S.yintiaoling* Kong, Zhang & Wang, sp. nov.; **8.***Amaurobiusdanba* Lu, Lin & Li, 2024; **9.***A.foping* Kong, Zhang & Wang, sp. nov.; **10.***A.pingwu* Kong, Zhang & Wang, sp. nov.; **11.***A.yaan* Lu, Lin & Li, 2024; **12.***A.yushen* Kong, Zhang & Wang, sp. nov.

Figs 1B–D , 7 , 8 , 9 渝神暗蛛

#### Type material.

***Holotype*** • ♂ (SWUC-T-AM-26-01): **China**, Chongqing Munic., Wuxi Co., Yintiaoling Nature Reserve, Qianzipa Station, Zhujiazhaizi Cave; 31°28′27″N, 109°47′9″E, elev. 1970 m; 22.9.2022; L.Y. Wang et al. leg.; ***Paratypes*** (6♂11♀): • 2♂2♀ (SWUC-T-AM-26-02 to 05), same data as holotype; • 3♂2♀ (SWUC-T-AM-26-06 to 10), Yintiaoling Nature Reserve, Guanshan Station, Yanzi Cave; 31°29′1″N, 109°43′49″E, elev. 2204 m; 22.9.2022; L.Y. Wang et al. leg.; • 1♂2♀ (SWUC-T-AM-26-11 to 13), Chenkou Co., Heyu Co., Xumu Vill., Lilaopo; 31°54′124″N, 109°3′0.7″E, elev. 1591 m; 12.8.2023; Z.S. Zhang et al. leg.; **Hubei** • 5♀ (SWUC-T-AM-26-14 to 18), Shennongjia, Hongping Town, Tianyan scenic area; 31°42’53″N, 110°27′42″E, elev. 2114 m; 22.9.2023; L.Y. Wang et al. leg.

#### Etymology.

The specific name is derived from the Chinese word ‘yu’ and ‘shen’, Yu (渝) is an abbreviation for Chongqing and Shen (神) is an abbreviation for Shennongjia (神农架).

#### Diagnosis.

The male of this new species resembles those of *A.yaan* Lin & Li, 2024 (Lu et al. 2024: 121, figs 4A, B, 5A, B, 8E, F) in that the males have similar median and retrolateral tibial apophyses, and the females have similar lateral teeth and spermathecae. However, the new species can be distinguished by the dorsal tibial apophysis (DTA) finger-like, larger than its prolateral branch (pb) (Figs 7C, E, 8C, E) (vs dorsal apophysis subequal in length to its prolateral branch). The female of this new species resembles those of *A.foping* sp. nov. (Fig. 5) in having similar elliptical spermathecae (S) and fertilization ducts (FD), but can be distinguished by the tip of lateral teeth (LT) pointed (Fig. 7F) (vs blunt); median plate about 2 times wider than long (Fig. 7F) (vs about as long as wide); copulatory ducts (CD) distinct (Fig. 7G) (vs inconspicuous).

#### Description.

**Male** holotype (Fig. 7A) total length 11.03. Carapace 5.54 long, 4.29 wide; opisthosoma 5.18 long, 4.09 wide. Carapace brownish. Eye sizes and interdistances: AME 0.22, ALE 0.26, PME 0.20, PLE 0.23; AME–AME 0.13, AME–ALE 0.38, PME–PME 0.34, PME–PLE 0.56, ALE–PLE 0.13. MOA 0.59 long, front width 0.56, back width 0.77. Clypeus height 0.22. Chelicerae with 4 promarginal and 3 retromarginal teeth. Legs yellowish brown. Leg measurements: I 15.42 (4.14, 5.43, 3.65, 2.20); II 14.59 (4.08, 5.20, 3.42, 1.89); III 12.79 (3.83, 4.37, 2.98, 1.61); IV 15.15 (4.22, 5.49, 3.70, 1.74). Opisthosoma oval, dorsum yellowish, with 6 brown chevrons extending posteriorly, venter yellowish brown.

***Palp*** (Fig. 7C–E). Tibia about 2 times longer than wide, with elongated RTA, originating near tip of tibia. DTA large, longer than wide, slightly curved dorsally, prolateral branch (pb) small, finger-like. Tegulum widest in middle part. Sperm duct visible in prolateral and retrolateral view. Tegular apophysis (TA) slightly raised. Median apophysis (MA) hook-like, located near center of tegulum. Conductor (C) membranous and sheet-like. Embolus originating prolaterally, short, flat and sharply pointed, with thin apex.

**Female** paratype (SWUC-T-AM-26-02, Fig. 7B) total length 13.66. Carapace 6.37 long, 4.74 wide; opisthosoma 7.11 long, 5.83 wide. Eye sizes and interdistances: AME 0.24, ALE 0.33, PME 0.21, PLE 0.27; AME–AME 0.14, AME–ALE 0.46, PME–PME 0.39, PME–PLE 0.73, ALE–PLE 0.19. MOA 0.76 long, front width 0.68, back width 0.85. Clypeus height 0.29. Legs yellowish brown. Leg measurements: I 14.22 (4.24, 4.97, 3.15, 1.86); II 13.37 (4.12, 4.76, 2.79, 1.70); III 11.55 (3.28, 4.34, 2.40, 1.53); IV 13.93 (4.13, 4.93, 3.33, 1.54). Chelicerae with 4 promarginal and 3 retromarginal teeth. Opisthosoma as in male.

***Epigyne*** (Fig. 7F, G). Epigynal plate 2 times wider than long. Median plate (MP) trapezoidal wider than long, with shallow excision on anterior margin medially; lateral teeth (LT) triangular, pointed. Spermathecae (S) elliptical, spaced by almost one width. Copulatory ducts (CD) tubular. Fertilization ducts (FD), originating posteriorly.

#### Variation.

Male (*N* = 7) total length 10.64–11.03, females (*N* = 11) total length 13.66–13.84.

#### Distribution.

China (Chongqing, Hubei) (Fig. 9).

## Supplementary Material

XML Treatment for Amaurobiidae

XML Treatment for Amaurobiinae

XML Treatment for Sinoamaurobius

XML Treatment for Sinoamaurobius baima

XML Treatment for Sinoamaurobius chengkou

XML Treatment for Sinoamaurobius guangwushanensis

XML Treatment for Sinoamaurobius songi

XML Treatment for Sinoamaurobius spinatus

XML Treatment for Sinoamaurobius wulongdongensis

XML Treatment for Sinoamaurobius yintiaoling

XML Treatment for Amaurobius

XML Treatment for Amaurobius foping

XML Treatment for Amaurobius pingwu

XML Treatment for Amaurobius yushen

## ﻿Additional information

### Use of AI

No use of AI was reported.

### Author ORCIDs

Jin-Xia Kong https://orcid.org/0009-0004-0956-7369

Yan-Nan Mu https://orcid.org/0000-0002-2504-673X

Ben-Chao Zhu https://orcid.org/0009-0007-1235-8308

Wei Lu https://orcid.org/0009-0005-3504-3777

Zhi-Sheng Zhang https://orcid.org/0000-0002-9304-1789

Lu-Yu Wang https://orcid.org/0000-0002-5250-3473

### Data availability

All of the data that support the findings of this study are available in the main text.
